# Overall Nutritional and Sensory Profile of Different Species of Australian Wattle Seeds (*Acacia* spp.): Potential Food Sources in the Arid and Semi-Arid Regions

**DOI:** 10.3390/foods8100482

**Published:** 2019-10-11

**Authors:** Kinnari J. Shelat, Oladipupo Q. Adiamo, Sandra M. Olarte Mantilla, Heather E. Smyth, Ujang Tinggi, Sarah Hickey, Broder Rühmann, Volker Sieber, Yasmina Sultanbawa

**Affiliations:** 1 Queensland Alliance for Agriculture and Food Innovations, Health and Food Sciences Precinct, Cooper Plains, Brisbane 4108, QLD, Australia; k.shelat@uq.edu.au (K.J.S.); o.adiamo@uq.edu.au (O.Q.A.); s.olartemantilla@uq.edu.au (S.M.O.M.); h.smyth@uq.edu.au (H.E.S.); 2 Australian National Fabrication Facility–Queensland Node, Australian Institute of Bioengineering and Nanotechnology, The University of Queensland, St. Lucia, Brisbane 4067, QLD, Australia; 3 Health Support Queensland, Queensland Health, Inorganic Chemistry, Forensic and Scientific Services, Coopers Plains, Brisbane 4108, QLD, Australia; ujang.tinggi@health.qld.gov.au; 4 Karen Sheldon Catering, PO Box 2351, Parap 0812, NT, Australia; sarah@karensheldoncatering.com.au; 5 Department of Chemistry of Biogenic Resources, Technical University of Munich, 94315 Straubing, Germany; broder.ruehmann@tum.de (B.R.); sieber@tum.de (V.S.)

**Keywords:** wattle seed species, nutritional profile, sensory profile, gel electrophoresis

## Abstract

Wattle seed *(Acacia* spp.*)* is a well-known staple food within indigenous communities in Australia. A detailed investigation of the overall nutritional and sensory profile of four abundant and underutilized *Acacia* species—*A.* *coriacea*, *A.* *cowleana*, *A.* *retinodes* and *A.* *sophorae*—were performed. Additionally, molecular weight of protein extracts from the wattle seeds (WS) was determined. The seeds are rich in protein (23–27%) and dietary fibre (33–41%). Relatively high fat content was found in *A. cowleana* (19.3%), *A. sophorae* (14.8%) and *A. retinodes* (16.4%) with oleic acid being the predominant fatty acid. The seeds contained high amounts of essential amino acids (histidine, lysine, valine, isoleucine and leucine). *A.* *coriacea* is rich in iron (43 mg/kg), potassium (10 g/kg) and magnesium (1.7 g/kg). Pentose (xylose/arabinose), glucose, galactose and galacturonic acids were the major sugars found in the four species. Raw seeds from *A. sophorae*, *A. retinodes* and *A. coriacea* have the highest protein molecular weight, between 50–90 kDa, 80 kDa and 50–55 kDa, respectively. There was variation in the sensory profile of the WS species. This study showed that the four WS species have good nutritional value and could be included in human diet or used in food formulations.

## 1. Introduction

As the world population increases and natural resources diminish, there has been a serious concern on available sustainable nutritious foods [1]. In addition, a majority of people from developing countries suffer from protein malnutrition, famine and different kinds of diseases due to inadequate food supply and poor quality food [2]. In order to meet these continued population growth and nutritional requirements, studies are required to examine and discover new sources of food. In the past few years, researchers have focused on the use of underutilized plant products as human food and animal feed [3,4,5].

The genus *Acacia*, commonly known as wattle, belongs to the family *Fabaceae* and it is a large group of woody species comprising of shrubs. *Acacia* subgenus *Phyllodineae* are naturally the most common *Acacia* species found in Australia and are among the most promising native leguminous plants [6,7]. These *Acacias* have been reported to exhibit significant potential to lower poverty in semi-arid regions of Africa [8,9]. Moreover, the seeds from various *Acacia* species, which were used traditionally as source of food by Australian Indigenous population, have been economically revived as food additives, such as emulsifying and flavouring agents [10,11,12]. *Acacia victoriae* Bentham is the most common species of *Acacia* with high water-soluble carbohydrates and protein contents and, thus, have been reported to have significant functional properties in food systems [13,14]. Additionally, *Acacia* plants have been frequently used to treat diseases, such as fever, leucorrhoea, throat infection, diarrhoea and haemoptysis [15].

However, several *Acacia* species which are also widely cultivated by indigenous people in different regions of Australia have not been fully utilized in food formulations or incorporated in human diets. These includes *A. coriacea* and *A. cowleana* which occurs throughout Northern Australia as well as *A. retinodes* and *A. sophorae* that are found in Southern and Southeastern Australia. These *Acacia* plants, particularly *A. retinodes*, are mainly used for gum production and ornamental purposes [16]. Nevertheless, information on the nutritive value of these *Acacia* species is sparse which may limit their use in foods. Therefore, this study investigated the overall nutritional value of seeds of these abundant and native Australian *Acacia* species. Furthermore, the seeds were roasted and the molecular weight profiles of protein extracts before and after treatment were examined. In addition, a preliminary sensory profiling of the four wattle seed species was carried out. This study will provide information on whether or not it is advisable to incorporate these seeds into the human diet and indicate the sensory characteristics of these species.

## 2. Materials and Methods

### 2.1. Materials

**Figure 1:**
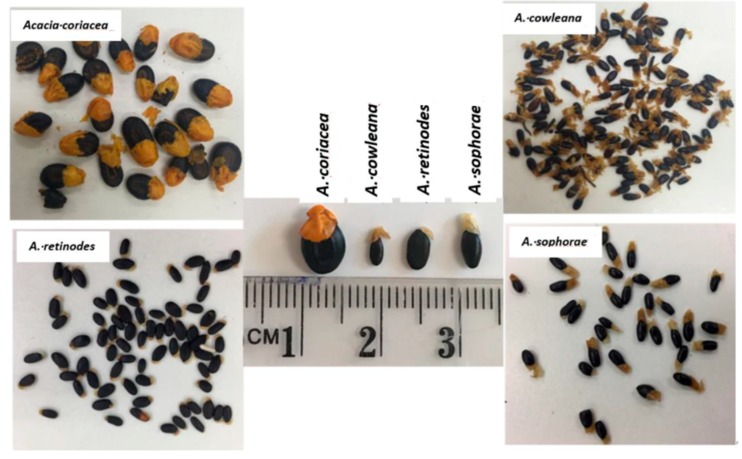
Four species of Australian wattle seeds—appearance and size comparison.

Mature seeds of four different species of Australian *Acacia* species (*A. coriacea*, *A. cowleana*, *A. retinodes* and *A. sophorae*) used in this study are shown in Figure 1. *A. coriacea* and *A. cowleana* were sourced from NATIF Australian Native Superfoods, Fruits Herbs Spices and Mixes, Victoria, Australia, and *A. retinodes* and *A. sophorae* were supplied by Valley Seeds Pty Ltd., Victoria, Australia. The seeds from each species were separately ground using a coffee grinder (power: 200 W, time: 30 s) and stored in the refrigerator until further analysis. Additionally, parts of the whole seeds were roasted at 180 °C for 5 min and used to determine the molecular weight profile of the seeds protein extracts for comparison with that obtained from raw seeds. All samples were analysed at least in duplicate and the average for each parameter was reported.

### 2.2. Proximate Analysis

The four different species of *Acacia* seeds were sent to Symbio Alliance Lab Pty Ltd., Eight Mile Plains, Queensland, Australia. A complete proximate analysis was performed at this accredited National Association of Testing Authorities (NATA) laboratory using AOAC [17] standard methods. The following analysis were measured: moisture (AOAC 925.10) by air oven with a measurement of uncertainty (MU) of ±15%, ash (AOAC 923.03), crude protein (AOAC 990.03) by Dumas combustion with a MU of ±10%, crude fat (AOAC 991.36) with a MU of ±15% and dietary fibre (985.29) with a MU of ±15%, carbohydrate and energy by calculation using information from the Food Standards Code.

### 2.3. Sugar Analysis

A combination of fast liquid chromatography coupled to UV and electrospray ionization trap detection (LC-UV-ESI-MS/MS) was used for the quantification of various sugars [18]. The hydrolysis was performed in duplicates by adding 6 mL of 2 M TFA to 12 mg of grounded *Acacia* seeds into 15 mL glass tubes. The tubes were incubated in a heating block (VLM GmbH, EC-Model, Heideblümchenweg, Bielefeld, Germany) for 90 min at 121°C. After cooling to room temperature, the hydrolysates were neutralized to pH ~8 by adding an aqueous solution of 3.2% NH_4_OH, since light alkaline conditions are required for the subsequent derivatization of monosaccharides. A 25 µL of neutralized hydrolysate supernatant were derivatized via the high throughput 1 phenyl-3-methyl-5-pyrazolone (HT-PMP) method [18]. The calibration standards were diluted with neutralized TFA-matrix to compensate the influence on the derivatization process. Each sample was derivatized in triplicates and the carbohydrate fingerprint was analysed.

### 2.4. Fatty Acid Profiles

About 1 g of finely chopped seed samples were taken for initial extraction with chloroform and methanol (2:1) followed by agitation at room temperature for 1 h and centrifuged for 5 min at 3500× *g*. Fatty acid profiling was performed at the School of Agriculture and Food sciences, University of Queensland laboratory. The GC-MS (Shimadzu QP2010, Shimadzu Coporation, Tokyo, Japan) was used at oven temperature of 100 °C, injector temperature 250 °C, total program time was 39 min, and helium used as the carrier gas. The inlet pressure used for gas chromatography was 0.4 kPa, at linear gas velocity of 42.7 cm/s, column (Restek stabilwax capillary column; 30 m × 0.25 mm ID × 0.5 µm film thickness) flow 1.10 mL/min with a split ratio of 1:1 and injection volume of 0.2 µL. For mass spectrometry, the ion source temperature used was 200 °C, the interface temperature was 250 °C and the mass range was 35–500 atomic mass units. Identification of the compounds was done by comparing their retention times and mass spectra with corresponding data from a standard food industry FAME Mix (Restek Corporation, Bellefonte, PA, USA).

### 2.5. Amino Acid Analysis

The samples (100 mg per replicate) were first hydrolysed with 6 M HCl at 110 °C for 24 h. As asparagine is hydrolysed to aspartic acid and glutamine to glutamic acid, the reported amount of these acids is the sum of those respective components. After hydrolysis, all amino acids were analysed at the Department of Molecular Science, Australian Proteome Analysis Facility, Macquarie University, NSW, Australia, using the Waters AccQTag Ultra chemistry on a Waters Acquity UPLC. Samples were analysed in duplicate and results are expressed as an average. The coefficient of variation (CV) of the UPLC analysis of amino acids was less than 5%.

### 2.6. Mineral Analysis

A detailed description of method used for mineral analysis is outlined as described by Carter et al. [19]. The ground seed samples were accurately weighed (0.3 g) into digestion Teflon vessels and concentrated nitric acid (4 mL) was added. The samples were digested using a microwave digestion system (MarsXpress, CEM, Matthews, NC, USA) programmed to three steps: step 1 (400 W power, 85 °C, 14 min), step 2 (800 W power, 110 °C, 20 min), and step 3 (1600 W power, 160 °C, 10 min) and the analysis was performed using inductively coupled plasma mass spectrometry (ICP-MS 7500a, Agilent, Tokyo, Japan) and optical emission spectrometry (ICP-OES, Varian Australia, VIC, Australia).

### 2.7. Sodium Dodecyl Sulphate-Polyacrylamide Gel Electrophoresis (SDS-PAGE)

The SDS-PAGE analysis of lyophilized protein extracts from raw and roasted wattle seeds were conducted at an accredited Protein Expression Facility (PEF), University of Queensland, St Lucia, Queensland, Australia. The extracts were resuspended in PBS to a final concentration of 2 mg/mL. Samples were loaded onto a 4–12% Bis-Tris SDS-PAGE gel and run under denatured and reduced conditions, except where noted. Analysis was performed using a Bio-Rad Chemi-Doc^TM^ XRS + imaging system.

### 2.8. Rapid Sensory Profiling

A sensory tasting session was carried out to develop sensory descriptors of four WS species: *A. coriacea*, *A. retinodes*, *A. sophorae* and *A. cowleana*. Twelve trained assessors (nine females; three males) with an average age of 50 years old participated in a two-hour session where they provided descriptors for aroma, flavour and aftertaste. For sensory evaluation the seeds of the four species were roasted in the oven for 7 min at 180 °C and ground when cooled. The wattle seeds were presented in two forms for sensory evaluation: as ground seeds on their own and the ground seeds mixed with semolina paste to make them more palatable for the assessors. One gram of ground seeds was presented for aroma assessment in a 30 mL plastic cup covered with a lid and labelled with a three digit blinding code. The ground seeds mixed with semolina where used to conduct a second assessment of aroma and to assess flavour and after taste. Each assessor was presented with 6 g of 1:20 ground wattle seeds and hydrated semolina mix in a 30 mL plastic cup covered with a lid and labelled with the same three digit blinding code used for the non-mixed sample. Green apple and water were used as palate cleansers. Upon completion of the session tasting component the panel leader facilitated session where sensory descriptors of each category for four wattle seed species were summarised and a consensus reached. This resulted in a preliminary sensory profile for each variety.

### 2.9. Statistical Analysis

The data were calculated using Microsoft Excel 2013 and the results are expressed as mean of the triplicate experiments unless otherwise stated. Statistical analysis of the data was done by one-way analysis of variance procedure (ANOVA) using SPSS software (Version 23.0 IBM Corporation, Armonk, NY, USA), and means comparison was done using Duncan’s multiple range test at *p* < 0.05.

## 3. Results and Discussion

### 3.1. Proximate Composition

As shown in Table 1, all of the species had low moisture content (less than 9%) which are comparable with the 6.9% recorded for *A. victoriae* Bentham [20] and the range (6.3–8.0%) for *A. tumida* and *A. colei* [8]. The four species of wattle seeds (WS) showed high protein content, ranging from 22.5% in *A. coriacea* to 27.5% in *A. retinodes*. The values for crude protein obtained in this study were higher than that of *A. victoriae* Bentham [20] but lower than those found in different subspecies of *A. saligna* (28.6–32.6%) [3]. However, the protein contents were within the range (23.4–34.1%) reported for *A. tumida* and *A. colei* [8]. The results indicate that WS can serve as a source of protein in the diets of the Aboriginal population in Australia. Moreover, it can be included in food formulations as a source of protein. The crude fat content varied among the four species with relatively high values observed in *A. cowleana* followed by *A. retinodes* while *A. coriacea* had the least value (9.8%). These results showed that *A. cowleana* seed would be a good source of energy due to its relatively high crude fat content. All the four species studied had similar ash contents (3.4–3.9%) which were comparable to that found in *A. victoriae* Bentham [20] and *A. tortilis* [21]. All seeds also showed high amounts of dietary fibre ranging from 33.7% in *A. cowleana* to 41.4% in *A. coriacea*. These values were higher than that recorded for *A. tortilis* [21] and *A. victoriae* Bentham [20]. Therefore, the seeds from these *Acacia* species can be considered as a good source of dietary fibre. Dietary fibre has a vital function in human nutrition by maintaining the health of the gastrointestinal tract, however, in excess may bind to iron and zinc, thereby lowering their bioavailability [22]. Regarding the carbohydrate content, the seeds have a range of 12.8–15.6% carbohydrate. These levels of carbohydrate were lower than that reported for *A. victoriae* Bentham [20] and *A. tumida* [2], due to the greater amounts of crude fat, fibre and protein in the seeds. Traditionally, *A. coriacea* is known to lower postprandial glucose level [23,24], higher dietary fibre and protein content could be the reason for these potential health benefits. This suggest that using wattle seed flour can be a healthier option. Therefore, the chemical composition of these wattle seed species was found to be nutritious and adding these seeds into the human diets will enhance the nutrition status.

### 3.2. Fatty Acid Composition

The fatty acid composition of the four species of wattle seed are presented in Table 2. The unsaturated fatty acids (UFA) constitute the bulk of the fatty acids, as in the case of certain edible legumes, such as peanut [25]. Palmitic acid (17.69–23.83%) was found to be the predominant saturated fatty acid (SFA) in all the seeds, followed by stearic acid (4.15–10.12%) with *A. coriacea* and *A. retinodes* having significantly (*p* < 0.05) higher values in the two fatty acids, respectively. Monounsaturated fatty acids (MUFA) contributed the larger percentage of the total UFA with values more than twice that of polyunsaturated fatty acids (PUFA) in *A. coriacea*, *A. sophorae* and *A. retinodes*, unlike in other legumes and wattle seed species [3,26]. The C18:1 was found to be the most abundant of all the MUFAs with similar values (50.82–57.57%) obtained in all the species except *A. cowleana* (36.24%) (*p* < 0.05). However, greatest (*p* < 0.05) amount of PUFA particularly linoleic acid was found in *A. cowleana* (34.77%) as compared to other species, with *A. sophorae* having significantly (*p* < 0.05) lowest PUFA value (7.17%). These four species have lower linoleic acids as compared to that of other legumes such as broad beans (48.3%), chickpeas (56.7%) and small lentils (52.3%) [26], as well as other WS species such as *A. saligna* [27] and *A. tortilis* [21]. Oils rich in linoleic acid can have an important role in lowering the levels of blood cholesterol [28], thus the oil obtained from *A. cowleana* is highly nutritious when consumed regularly as a part of diet. Moreover, only trace amount of *n*-3 fatty acids was identified in all the seeds. Overall, the high degree of unsaturation found in all the four WS species agrees with those of common vegetable oils indicating that WS composite flour can be a healthy option for human consumption.

### 3.3. Amino Acid Composition

The essential amino acid analysis of four different species of WS showed all four of them contained about 13–15% of glutamic acid, followed by about 9–11.5% of aspartic acid, 6.8–7.4% of lysine, 8.0–8.6% of leucine and about 7% of arginine, serine and alanine (Table 3). Moreover, methionine is the limiting amino acids found in all four seeds and this agrees with that reported in other *Acacia* seed species [21,22]. This is the first time that a complete detailed analysis of all essential amino acids of these four different species of wattle seeds have been investigated. Due to high amounts of protein in all these species, it is important to look at different types of amino acids available in these seeds. It is observed that the glutamic acid content is slightly lower than that of about 18% in mung bean flour [29]. The amount of lysine in all four seeds is comparable to that of soybean [30]. The amount of arginine is very similar to that of winged bean and soy beans [31]. Presence of high amount of arginine potentially can help to increase physiological pool of L-arginine and may have positive impact on cardiovascular health [32]. Comparing the amino acid profile of the seed proteins with FAO reference pattern [33] can be used to justify the potential food value of the proteins. The amino acid profile of the four WS species showed that histidine, lysine, valine, isoleucine and leucine had higher levels than those listed in FAO/WHO reference pattern.

### 3.4. Mineral Composition

Table 4 outlines a detailed mineral analysis performed on four different species of Australian WS. As highlighted in the table, all four species are good sources of important essential minerals such as iron, potassium, magnesium, calcium and zinc. Potassium is the most abundant element in all the WS species with *A. coriacea* having significantly (*p* < 0.05) highest value (11,000 mg/kg dry weight (DW)). The results obtained agrees with that reported for different WS species [8,21,22]. A significantly (*p* < 0.05) higher iron content (195.0 mg/kg DW) was found in *A. sophorae* as compared to other species of WS, showing it is a good source of iron considering the Australian recommended dietary allowance (RDA) of iron is 7 and 12–16 mg/day for men and women during pregnancy, respectively [34,35]. Moreover, heavy metals, such as Hg, are less than 0.005 mg in all four WS species, indicating the use of all these four species of WS is safe for human consumption. As stated in the Food Standards Australia New Zealand (FSANZ), Standard 1.4.1 for contaminants and Natural Toxicants, the maximum level of Pb in legumes is set at 0.2 mg/kg and cadmium in rice is 0.1 mg/kg. The amount of Pb in all four WS species is within this range and in case of *A. sophorae* and *A. cowleana*, they have significantly (*p* < 0.05) higher values (0.1 and 0.175 mg/kg DW, respectively) as compared to other species, but the values still complied with the food standards code [36]. Based on the above results, the WS are a good source for minerals and thus can be incorporated into foods, such as commercial baked products, that are deficient in minerals to enhance their nutritional properties. Further studies should be carried out to investigate the bioavailability of the essentials minerals, particularly potassium in the WS species.

### 3.5. Sugar Profile

The results in Table 5 show that the monosaccharides are the major kind of sugar present in all the four WS species. Pentose sugars (xylose/arabinose) were the predominant monosaccharides found in the seeds and these were followed by galactose and glucose, while a small amount of mannose and fucose were present. Among the species, *A. sophorae* has a significantly (*p* < 0.05) higher amount of pentose sugars (84.2%), while significantly (*p* < 0.05) higher glucose and galactose contents were found in both *A. sophorae* and *A. retinodes*. Moreover, the seeds are rich in galacturonic acid but contain lesser amounts of rhamnose. All these sugars were present in substantial amount in all the species, particularly in *A. retinodes*, *A. sophorae* and *A. coriacea* as compared to *A. cowleana*. To the best of our knowledge, this study was the first to report the sugar composition present in wattle seeds. Overall, the results revealed that these wattle seed species have high amount of reducing sugar and this may be due to action of endogenous enzymes involved in hydrolysing the stored carbohydrate during maturation and storage.

### 3.6. SDS-PAGE Profile of Soluble Proteins Extracted from Raw and Roasted Wattle Seed Species

**Figure 2:**
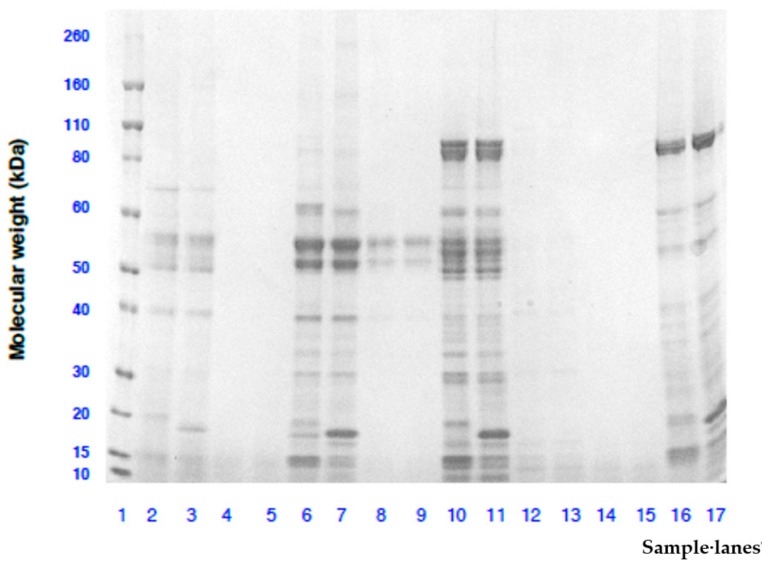
Molecular weight analysis by SDS-PAGE of protein extracts from raw and roasted wattle seeds species. Lanes 1: Novex Sharp Pre-stained molecular weight ladder; Lanes 2, 3, 4 and 5: Raw and reducing, raw and non-reducing, roasted and reducing, roasted and non-reducing *A. cowleana*, respectively; Lanes 6, 7, 8 and 9: Raw and reducing, raw and non-reducing, roasted and reducing, roasted and non-reducing *A. coriacea*, respectively; Lanes 10, 11, 12 and 13: Raw and reducing, raw and non-reducing, roasted and reducing, roasted and non-reducing *A. sophorae*, respectively; Lanes 14, 15, 16 and 17: Roasted and reducing, roasted and non-reducing, raw and reducing, raw and non-reducing *A. retinodes*, respectively.

Figure 2 shows the SDS-PAGE gel electrophoretograms of extracts from four species of raw and roasted WS obtained under non-reducing and reducing conditions. For raw WS extracts in the four species, similar protein bands appeared under reducing and non-reducing conditions but protein molecules smaller than 18 kDa were broken down under reducing condition probably due to reduction in disulphide bonds. Moreover, more protein bands were found in raw *A. sophorae* (lanes 10 and 11) followed by *A. coriacea* (lanes 6 and 7) and *A. retinodes* (lanes 16 and 17) while *A. cowleana* (lanes 1 and 2) showed faint protein bands. Overall, the results suggest that water soluble proteins from raw WS species were mainly polypeptides with less than 80 kDa molecular weights for *A. sophorae* and *A. retinodes* and 55 kDa for *A. coriacea*. This differs from previous study on different wattle seed species where the molecular weight of the protein was found to be lower than 66 kDa [13,27]. After roasting, no protein band was visible in the four wattle seed species with the exception of only two faint protein bands observed in *A. coriacea* (lanes 8 and 9) at 55 kDa. The results showed that the heat treatment had caused the soluble proteins in WS to be degraded into fragments with molecular weight smaller than 10 kDa. A similar effect of thermal treatment on protein had been reported in previous studies on wattle seeds [27] and peanuts [37].

### 3.7. Rapid Sensory Profiling

The results of the rapid sensory profiling presented in Table 6 showed a wide diversity of sensory characteristics for the four WS species. The summary of the descriptors provided by the trained panel showed that, while all four species had savoury notes, certain descriptors characterised each species. *A. cowleana,* for instance, was perceived as a complex cultivar where the broth, popcorn and savoury notes where more intense for aroma and flavour. *A. coriacea* was distinguished by some confectionary and tropical fruit notes. *A. sophorae* was characterised by more savoury and grassy notes but not very intense. Cultivar *A. retinodes* was characterised by gravy, lemony earthy and nutty notes. Previous preliminary studies in *A. victoriae* have shown that this wattle seed cultivar has a savoury note as the four WS species in this study but also its own nutty and wheat biscuit notes [38]. The informal sensory component of this study has shown diversity in sensory profiles between WS seed species, opening the door for different applications of WS species in the food industry. Future work could investigate more rigorously the sensory differences between these four species and other species of the WS, as well as investigate effects of roasting and grinding on flavour.

## 4. Conclusions

This study shows that the four species of wattle seeds are good source of protein and dietary fibre. In addition, the seeds can be considered as a potential dietary source of major and minor minerals, essential amino acids and oleic acids, thus, the seeds have promising nutritional profiles. Nevertheless, further studies should be carried out to investigate the impact of processing on these nutrients and also to evaluate the anti-nutritional components present in these species before and after processing.

## Figures and Tables

**Table 1:** Proximate composition of four different species of wattle seeds (%, dry weight).

Composition	A. cowleana *	*A. coriacea*	*A. sophorae*	*A. retinodes*	*A. victoriae* [19]
Moisture (%)	5.3	8.6	7.5	5.6	6.9
Crude protein (%)	23.0	22.5	22.7	27.5	17.5
Crude fat (%)	19.3	9.8	14.8	16.4	3.2
Ash (%)	3.4	3.9	3.5	3.7	3.5
Dietary fibre (%)	33.7	41.4	36.0	34.0	29.4
Non-fibre carbohydrate (%)	15.2	13.7	15.6	12.8	67.5
Energy (kJ)	1634.0	1310.0	1485.0	1563.0	1384.0

Values are the means of triplicate analyses. * Estimated measurement of uncertainty (MU) was calculated at 95% confidence interval. *A. cowleana*: *Acacia cowleana.*

**Table 2:** Fatty acid profile of four different species of wattle seeds expressed as percentage (± SD) of the total fatty acid profile as determined by FAME GC-MS analysis.

Fatty acid (%)	*A. cowleana*	*A. coriacea*	*A. sophorae*	*A. retinodes*
Lauric acid (C12:0)	0 ^b^	0 ^b^	0.56 ± 0.03 ^a^	0 ^b^
Myristic acid (14:0)	0 ^b^	0 ^b^	1.33 ± 0.07 ^a^	0 ^b^
Palmitic acid (C16:0)	21.3 ± 0.35 ^b^	23.8 ± 0.35 ^a^	21.6 ± 0.77 ^b^	17.7 ± 0.11 ^c^
Stearic acid (C18:0)	4.15 ± 0.05 ^c^	4.36 ± 0.17 ^c^	7.51 ± 0.31 ^b^	10.1 ± 0.21 ^a^
Arachidonic acid (C20:0)	0.99 ± 0.05 ^a^	1.07 ± 0.10 ^a^	0.86 ± 0.03 ^b^	0.95 ± 0.06 ^ab^
Behenic acid (C22:0)	2.49 ± 0.07 ^a^	1.80 ± 0.13 ^b^	0.34 ± 0.03 ^c^	1.02 ± 0.04 ^b^
Lignoceric acid (C24:0)	0 ^b^	0 ^b^	0.16 ± 0.01 ^a^	0 ^b^
Total SFA	28.9 ± 0.52 ^c^	31.0 ± 0.75 ^ab^	32.4 ± 1.25 ^a^	29.8 ± 0.42 ^bc^
Palmitic acid (C16:1)	3.40 ± 0.03 ^b^	0.45 ± 0.01 ^c^	3.84 ± 0.27 ^a^	0.46 ± 0.03 ^c^
Oleic acid (C18:1)	32.8 ± 0.43 ^c^	50.8 ± 0.26 ^b^	57.6 ± 0.49 ^a^	50.1 ± 1.34 ^b^
Ecosanoic acid (C20:1)	0 ^c^	0 ^c^	0.37 ± 0.01 ^b^	1.57 ± 0.02 ^a^
Erucic acid (C22:1)	0 ^b^	0 ^b^	0 ^b^	0.52 ± 0.01 ^a^
Total MUFA	36.2 ± 0.46 ^c^	51.3 ± 0.27 ^b^	61.8 ± 0.77 ^a^	52.7 ± 1.4 ^b^
Linoleic acid (C18:2)	34.3 ± 0.08 ^a^	17.4 ± 0.33 ^b^	6.76 ± 0.75 ^c^	16.0 ± 1.64 ^b^
Linolenic acid (C18:3)	0.49 ± 0.02 ^b^	0.27 ± 0.04 ^c^	0.41 ± 0.04 ^b^	1.61 ± 0.08 ^a^
Total PUFA	34.8 ± 0.10 ^a^	17.7 ± 0.73 ^b^	7.17 ± 0.79 ^c^	17.6 ± 1.72 ^b^

SFA: Saturated fatty acids; MUFA: Monounsaturated fatty acids; PUFA: polyunsaturated fatty acids. Means not sharing the same superscript’s letters (a, b, c, d) in a row are significantly different at *p* < 0.05 as assessed by Duncan’s multiple range tests.

**Table 3:** Amino acid (g/100 g dry weight) profile of four different species of wattle seeds.

Amino acid	*A. cowleana*	*A. coriacea*	*A. sophorae*	*A. retinodes*	FAO/WHO References [32]
Essential amino acids					
Histidine	2.8	2.6	4.3	2.4	1.9
Threonine	4.1	4.0	4.2	4.3	3.4
Lysine	7.0	6.8	7.4	7.0	5.8
Tyrosine	2.0	2.1	2.1	2.2	6.3
Methionine	0.4	0.4	0.3	0.3	
Valine	5.9	5.8	6.0	6.2	3.5
Isoleucine	4.4	4.2	4.4	4.3	2.8
Leucine	8.4	8.0	8.6	8.5	6.6
Phenylalanine	3.6	3.3	3.5	3.5	
Non-essential amino acids					
Serine	7.0	7.7	7.1	7.1	
Arginine	6.0	7.1	6.0	6.3	
Glycine	9.7	10.6	9.5	10	
Aspartic acid	10.5	11.5	9.4	10.7	
Glutamic acid	15.3	13.3	14.2	14.2	
Alanine	7.3	6.7	7.1	7.0	
Proline	5.7	5.8	6.0	6.0	

Results are expressed as the mean of duplicate experiments.

**Table 4:** Mineral analysis of four different species of wattle seeds.

Minerals (mg/kg DW)	*A. cowleana*	*A. coriacea*	*A. sophorae*	*A. retinodes*
Major				
Ca	2300 ± 0.0 ^c^	4300 ± 141.4 ^a^	2600 ± 424.3 ^c^	3150 ± 212.1 ^b^
K	8700 ± 0.0 ^b^	11000 ± 0.0 ^a^	7300 ± 141.4 ^c^	9050 ± 495.0 ^b^
Mg	1700 ± 0.0 ^b^	2350 ± 70.7 ^a^	1700 ± 0.0 ^b^	2400 ± 141.4 ^a^
Na	<20 ^c^	<20 ^c^	1100 ± 0.0 ^a^	940 ± 70.7 ^b^
P	1700 ± 0.0 ^b^	2350 ± 70.7 ^a^	2300 ± 0.0 ^a^	2300 ± 141.4 ^a^
Trace				
Co	0.1 ± 0.0 ^c^	0.04 ± 0.0 ^b^	0.365 ± 0.0 ^a^	0.095 ± 0.0 ^c^
Cr	2.1 ± 0.2 ^a^	0.8 ± 0.1 ^c^	1.95 ± 0.2 ^a^	1.15 ± 0.2 ^b^
Cu	4.8 ± 0.1 ^c^	5.0 ± 0.0 ^c^	8.65 ± 0.2 ^a^	7.2 ± 0.1 ^b^
Fe	75.0 ± 0.0 ^b^	50.5 ± 0.7 ^b^	195.0 ± 35.4 ^a^	49.5 ± 0.7 ^b^
Mn	14.0 ± 0.0 ^c^	14.0 ± 0.0 ^c^	46.5 ± 2.1 ^b^	130.0 ± 0.0 ^a^
Mo	0.9 ± 0.2 ^c^	0.84 ± 0.1 ^c^	1.75 ± 0.1 ^b^	2.0 ± 0.0 ^a^
Se	0.9 ± 0.0 ^a^	0.68 ± 0.0 ^b^	0.165 ± 0.0 ^c^	0.34 ± 0.0 ^d^
Zn	24.5 ± 0.7 ^b^	23.0 ± 0.0 ^c^	21.0 ± 0.0 ^d^	34.0 ± 0.0 ^a^
Other minerals				
Al	42.0 ± 1.4 ^b^	16.5 ± 2.1 ^c^	77.5 ± 3.5 ^a^	3.75 ± 0.4 ^d^
As	ND	<0.005 ^b^	0.87 ± 0.2 ^a^	<0.005 ^b^
Ba	3.2 ^c^	49.0 ± 1.4 ^a^	1.0 ± 0.0 ^d^	7.15 ± 0.1 ^b^
Cd	<0.005 ^c^	<0.005 ^c^	0.054 ± 0.0 ^a^	0.011 ± 0.0 ^b^
Hg	<0.005 ^a^	<0.005 ^a^	<0.005 ^a^	<0.005 ^a^
Ni	2.7 ± 0.1 ^c^	1.1 ± 0.0 ^d^	3.8 ± 0.0 ^a^	3.05 ± 0.1 ^b^
Pb	0.1 ± 0.0 ^ab^	0.074 ± 0.0 ^b^	0.175 ± 0.1 ^a^	0.0085 ± 0.0 ^b^
Sb	ND	0.01 ± 0.0 ^a^	<0.01 ^a^	<0.01 ^a^
Sn	<0.05 ^b^	0.85 ± 0.1 ^a^	<0.05 ^b^	<0.05 ^b^
Sr	19.0 ± 0.0 ^b^	24.0 ± 1.4 ^a^	5.9 ± 0.1 ^d^	13.0 ± 0.0 ^c^
V	0.1 ± 0.0 ^b^	0.035 ± 0.0 ^b^	1.65 ± 0.4^a^	0.01 ± 0.0^b^

ND: not detected. Means not sharing the same superscript’s letters (a, b, c, d) in a row are significantly different at *p* < 0.05 as assessed by Duncan’s multiple range tests.

**Table 5:** Sugar profile of four different species of wattle seeds.

Sugar Composition (%)	*A. cowleana*	*A. coriacea*	*A. sophorae*	*A. retinodes*
Glucose	22.1 ± 1.30 ^c^	37.8 ± 1.01 ^b^	44.4 ± 1.60 ^a^	43.9 ± 2.16 ^a^
Mannose	4.17 ± 0.39 ^b^	4.05 ± 0.31 ^b^	4.47 ± 0.21 ^b^	5.48 ± 0.57 ^a^
Galactose	34.2 ± 1.30 ^c^	57.6 ± 2.23 ^b^	65.5 ± 3.35 ^a^	67.3 ± 2.91 ^a^
Galacturonic acid	30.3 ± 1.28 ^c^	51.7 ± 3.68 ^a^	44.1 ± 0.55 ^b^	51.3 ± 1.50 ^a^
Rhamnose	4.98 ± 0.32 ^c^	7.90 ± 0.53 ^a^	5.40 ± 0.32 ^c^	5.82 ± 0.19 ^b^
Fucose	2.77 ± 0.29 ^a^	2.43 ± 0.35 ^a^	2.63 ± 0.28 ^a^	2.75 ± 0.38 ^a^
Xylose/Arabinose	79.7 ± 3.43 ^b^	71.5 ± 4.84 ^c^	84.2 ± 2.24 ^a^	78.4 ± 1.08 ^b^

Means not sharing the same superscript’s letters (a, b, c, d) in a row are significantly different at *p* < 0.05 as assessed by Duncan’s multiple range tests.

**Table 6:** Rapid sensory profiling of four different species of wattle seeds.

Characteristics	*A. retinodes*	*A. cowleana*	*A. coriacea*	*A. sophorae*
Aroma ground powder	Mushroom, gravy, onion, coffee, chocolate, roasted nuts, lemon	Spicy, curried, savoury, popcorn, burnt toast, savoury jam, chemical fish stock.	Savoury, spinach, chocolate, sweet, fairy floss, tropical fruit, water of boiled vegetables	Yeasty (vegemite), meaty, burnt tyres, roasted onion
Aroma ground powder in semolina	Earthy, grassy, musty, pepper, oily peanut butter	Smoky, broth, lentil, turmeric, chemical	Cinnamon, dairy	Buttery, popcorn, big change from peppery, meaty to earthy.
Flavour	Bitter, nutty, leaves, earthy, peanut, sesame paste	Intense, bitter, burnt, peppery, chives, onion salt, roasted chicken stuffing, dried legumes.	Fruity, almost coffee, tahini, mashed vegetables, cereal, not very intense	Vegemite, nutty, savoury, slight grassy, onion
After taste	Grass, barnyard, smoky, savoury, cucumber.	Bitter, pepper, very lingering	Spinach, not very lingering	Mild, grassy, bitter, savoury
