# Correction: Shelat, K.J.; et al. Overall Nutritional and Sensory Profile of Different Species of Australian Wattle Seeds (*Acacia* spp.): Potential Food Sources in the Arid and Semi-Arid Regions. *Foods* 2019, *8*, 482

**DOI:** 10.3390/foods10030650

**Published:** 2021-03-19

**Authors:** Kinnari J. Shelat, Oladipupo Q. Adiamo, Sandra M. Olarte Mantilla, Heather E. Smyth, Ujang Tinggi, Sarah Hickey, Broder Rühmann, Volker Sieber, Yasmina Sultanbawa

**Affiliations:** 1 Queensland Alliance for Agriculture and Food Innovations, Health and Food Sciences Precinct, Cooper Plains, Brisbane, QLD 4108, Australia; k.shelat@uq.edu.au (K.J.S.); o.adiamo@uq.edu.au (O.Q.A.); s.olartemantilla@uq.edu.au (S.M.O.M.); h.smyth@uq.edu.au (H.E.S.); 2 Australian National Fabrication Facility–Queensland Node, Australian Institute of Bioengineering and Nanotechnology, The University of Queensland, St. Lucia, Brisbane, QLD 4067, Australia; 3 Health Support Queensland, Queensland Health, Inorganic Chemistry, Forensic and Scientific Services, Coopers Plains, Brisbane, QLD 4108, Australia; ujang.tinggi@health.qld.gov.au; 4 Karen Sheldon Catering, P.O. Box 2351, Parap, NT 0812, Australia; sarah@karensheldoncatering.com.au; 5 Department of Chemistry of Biogenic Resources, Technical University of Munich, 94315 Straubing, Germany; broder.ruehmann@tum.de (B.R.); sieber@tum.de (V.S.)

The authors wish to make the following correction to the paper [1]:

1. The title of Table 3 “Amino acid (g/100 g dry weight) profile of four different species of wattle seeds” should be changed to “Amino acid (mol% dry weight) profile of four different species of wattle seeds”.
2. The title of first column in Table 5 “Sugar composition (%)” should be changed to “Sugar composition (mg/g biomass)”.

The authors would like to apologize for any inconvenience caused to the readers by these changes. The errors do not significantly change the conclusions to the paper. The published version will be updated on the article webpage, with a reference to this correction notice.
